# Cell survival after DNA damage in the comet assay

**DOI:** 10.1007/s00204-021-03164-3

**Published:** 2021-10-05

**Authors:** Ezgi Eyluel Bankoglu, Carolin Schuele, Helga Stopper

**Affiliations:** Institute of Pharmacology and Toxicology, University of Wuerzburg, Versbacher Straße 9, 97078 Wuerzburg, Germany

**Keywords:** DNA damage, DNA repair, Cell death and comet assay

## Abstract

The comet assay is widely used in basic research, genotoxicity testing, and human biomonitoring. However, interpretation of the comet assay data might benefit from a better understanding of the future fate of a cell with DNA damage. DNA damage is in principle repairable, or if extensive, can lead to cell death. Here, we have correlated the maximally induced DNA damage with three test substances in TK6 cells with the survival of the cells. For this, we selected hydrogen peroxide (H_2_O_2_) as an oxidizing agent, methyl methanesulfonate (MMS) as an alkylating agent and etoposide as a topoisomerase II inhibitor. We measured cell viability, cell proliferation, apoptosis, and micronucleus frequency on the following day, in the same cell culture, which had been analyzed in the comet assay. After treatment, a concentration dependent increase in DNA damage and in the percentage of non-vital and apoptotic cells was found for each substance. Values greater than 20–30% DNA in tail caused the death of more than 50% of the cells, with etoposide causing slightly more cell death than H_2_O_2_ or MMS. Despite that, cells seemed to repair of at least some DNA damage within few hours after substance removal. Overall, the reduction of DNA damage over time is due to both DNA repair and death of heavily damaged cells. We recommend that in experiments with induction of DNA damage of more than 20% DNA in tail, survival data for the cells are provided.

## Introduction

The comet assay is a relatively simple method for quantification of DNA damage. Its application has increased tremendously and it is used in many areas of research as well as in mutagenicity testing for approval of pharmaceuticals and chemicals (Koppen et al. 2017). As an in vitro assay, it is applied mostly in the prescreening phase of substance development or in basic research. The application in vivo on rodent tissue is based on an OECD guideline (OECD 2016) and is suitable for detection of tissue specific genomic damage. In human biomonitoring, elevated genomic damage may be attributed to mutagen exposure, endogenous factors or disease situations. Ongoing research aims to identify individuals with elevated disease risk among an exposed group or a patient group to aid in the decision for further diagnostic or medical interventions (e.g. Anderson et al. 2019; Azqueta et al. 2020)).

These are all applications of high practical relevance. However, the interpretation of DNA damage as seen in the comet assay has not been discussed in depth recently. On the one hand, the detected DNA damage is in principle repairable (Collins 2004) and in fact, recent attempts in the area of human biomonitoring focus on using variations of the comet assay as a DNA repair assay to determine individual repair capacity (Azqueta et al. 2019; Valdiglesias et al. 2020). On the other hand, heavily damaged cells may die through apoptosis, necrosis or other types of cell death. These cellular fates are of opposite consequence since remaining damage or incorrect repair attempts may lead to mutations and malignant cell transformation, while cell death may eliminate such cells from the body as one of the many defense mechanisms against cancer. So far, it is not known which amount of DNA damage as seen in the comet assay is repairable and which amount causes cell death. Such knowledge would help in the interpretation of comet assay data.

Often, the disappearance of an induced DNA damage in the comet assay over time is interpreted as DNA repair (e.g. (Nickson and Parsons 2014)). However, the disappearance of highly damaged cells due to cell death may pose an alternative explanation for some experimental situations. Another way to detect presumable DNA repair in the comet assay is to use repair inhibitors, which increase the overall detectable amount of DNA damage. For example, the polymerase inhibitor aphidicolin increases the observable damage because cells can still cut out the lesions but not fill the gaps (Bankoglu et al. 2021). In this comet assay variation, interpretation may be complicated by the fact that aphidicolin may itself cause additional cell death and that it also affects DNA-replication. A not so clear relationship between aphidicolin-mediated increased DNA damage in the comet assay and DNA repair was also observed in an earlier study (Bausinger et al. 2015) which found that despite clearly measurable increase of DNA damage due to presumed excision of benzo(a)pyrene DNA adducts in human lymphocytes, there was no significant reduction of the adducts detectable in HPLC-analysis. On the other side, a reduction of adducts was measured by HPLC in A549 cells, while the comet assay did not show excision activity.

In our recent work, we have found that in frozen peripheral human lymphocytes, DNA damage increased in the comet assay within the first few hours after thawing and then decreased over the next 16 h, paralleled by a reduction of live/viable cells. Since G_0_ or G_1_ phase lymphocytes are not thought to exhibit much DNA repair activity (Bausinger et al. 2015), the death of heavily damaged cells could explain the reduced overall damage (Bankoglu et al. 2021).

Therefore, it is important to investigate the further fate of cells that are found positive in the comet assay. For this, we have used the oxidizing agent hydrogen peroxide (H_2_O_2_), the alkylating agent methyl methanesulfonate (MMS), and the topoisomerase II inhibitor etoposide, to represent three different mechanisms of action for induction of DNA damage measurable in the comet assay. We have investigated the time course of induction and reduction of DNA damage and have measured cell proliferation, viability, apoptosis and micronucleus frequency on the following day to relate the amount of DNA damage to the further fate of the cells.

## Materials and methods

### Materials

GelRed was obtained from Biotrend (Köln, Germany). Normal melting point agarose, dimethyl sulfoxide (DMSO), sodium hydroxide, and fully frosted slides were from Carl Roth (Karlsruhe, Germany). Low melting point agarose, RPMI 1640 medium, _L_-glutamine, sodium pyruvate, penicillin (100 µg/ml), streptomycin (1 mM), methyl methane sulfonate, hydrogen peroxide, and etoposide were from Sigma–Aldrich (Steinheim, Germany). Fetal calf serum was obtained from Anprotec (Bruckberg).

### Methods

#### Cell culture

TK6 cells were cultured in RPMI 1640 medium supplemented with 10% (v/v) fetal calf serum, 1% (w/v) _L_-glutamine, 1% (w/v) sodium pyruvate, and 0.4% (w/v) antibiotic (penicillin/streptomycin) in an incubator with 5% CO_2_ at 37 °C. One day prior to the experiment, 350.000 cells were seeded in a 6-well-plate. Next day, TK6 cells were treated with test substances (methyl methane sulfonate, MMS: 0–100–150–200–250–300 µM; hydrogen peroxide, H_2_O_2_: 0–20–40–60–80–100 µM, and etoposide, eto: 0–0.1–0.5–1–2.5–5 µM). Treatment duration for each substance was determined after performing an alkaline comet assay with a middle concentration of substance over time (0.5 to 20 h). The time points, which gave the maximum damage was selected for testing various concentration of these substances.

#### Alkaline comet assay

One ml of TK6 cell suspension was used for performing comet assay. For single gel format, cell suspension was centrifuged at 400×*g* for 5 min. Then supernatant was discarded and cells were resuspended in remaining medium. 20 µl of this cell suspension was used for mixing with 180 μl of pre-warmed low melting point agarose (0.5%). For 12-minigel format, 20 μl of cell suspension was directly mixed (without centrifugation step) with 180 μl of pre-warmed low melting point agarose (0.5%) at 37 °C. Subsequently for single gel and for minigel, 45 µl and 5 µl of these mixture was placed on a fully frosted slides that was coated with 1.5% of normal melting point agarose. After solidification of the gels, slides were dipped into a cold lysis solution (1% Triton X-100, 10% dimethyl sulfoxide and 89% lysis buffer containing 10 mM Tris, 2.5 M NaCl and 100 mM Na_2_EDTA with pH 10) for an hour. After lysis, slides were placed in a horizontal electrophoresis chamber filled with cold alkaline solution (1 mM Na_2_EDTA, 300 mM NaOH, pH > 13) and incubated for 20 min in the dark for DNA unwinding and then electrophoresis was performed (1 V/cm, 20 min). The slides with single gel was neutralized in PBS for 5 min and then dehydrated in ice-cold methanol for 5 min. The slides with minigels were washed in PBS and then in bidistilled water each for 10 min. For dehydration of minigels, slides were placed in 70% ethanol for 15 min and then in 100% ethanol for 30 min. After air-drying, all samples were stained with GelRed for scoring. The percentage of DNA in tail was scored using Komet6 software in 100 random nuclei (50 per replicate agarose gels) per sample.

#### Viability test

Cell viability was performed at the time of cell harvest for comet assay. After harvesting 1 ml cell suspension for comet assay, medium was changed for the remaining cells and cytochalasin B (1.5 µg/ml) was added to differentiate the cell death and proliferation. After the addition of cytochalasin B, viability of these cells were scored at 3 h and at 20 h. For viability test, 35 µl of cells suspension was mixed with 15 µl of staining solution (2 µl GelRed stock solution and 12 µl fluorescein diacetate (FDA, 5 mg/ml in acetone) in 2 ml PBS) and then 15 µl of this mixture was placed on a microscope slides and covered with a cover slip. FDA is activated to exhibit green fluorescence by cytosolic esterases in intact cells, while GelRed can only enter cells with compromised membrane integrity. In total 200 cells (red and green stained) per slide from each replicate were scored with an Eclipse 55i microscope (Nikon GmbH, Dusseldorf, Germany) at 200-fold magnification using FITC filter. The proportion of vital cells to dead cells was determined.

#### Cell count

In parallel to viability test, the number of cells were determined using a cell-counting chamber. To ensure an even cell distribution, samples were mixed by pipetting up and down and then 10 µl of cell suspension was pipetted into the well of counter chamber and then cells were counted using a hand counter from all four sets of squares. The average cell count from all four sets of squares were taken and then multiply by 10,000 and the volume of the medium to determine the number of cells in total.

#### Micronucleus test

The TK6 cells were incubated with the test substances for 4 h. After the incubation, medium was removed and fresh medium with cytochalasin B (1.5 µg/ml) was added for 20 h. Next day, cells were harvested and brought onto glass slides by cytocentrifugation and fixed in ice-cold methanol for 2 h. The slides were then stained with GelGreen staining solution (1% stock solution in water) and subsequently washed with PBS and mounted for microscopy. Scoring was done at a Nikon Eclipse TE 2000-E microscope with 400-fold magnification. The number of mononucleated (MoN), binucleated (BN), multinucleated (MuN), mitotic, and apoptotic cells were scored in 1000 cells on each slide and replicate per sample. The frequency of micronucleated cells was scored in 1000 binucleated cells on each slide of replicate per sample. The percentage of healthy proliferating cells were evaluated by multiplication of BN and MuN cells in 1000 cells from all categories (MoN, BN, MuN, mitotic, and apoptotic). The cytokinesis block proliferation index (CBPI) was calculated according to the following formula:

$${\varvec{C}}{\varvec{B}}{\varvec{P}}{\varvec{I}}=\frac{(\left(1\mathrm{ x MoN}\right)+\left(2 x BN\right)+\left(3 x MuN\right))}{(\mathrm{MoN}+\mathrm{BN}+\mathrm{MuN})}$$

#### Data analysis and statistics

Graphics were drawn using GraphPad Prism 9 software and statistical analysis was done using GraphPad Prism 9 software. Data are represented as mean ± sd of three independent experiments. The multiple comparison test Fisher's LSD was conducted following to one-way ANOVA to determine the significance between individual groups. Spearman correlation test was utilized for investigating the correlation between DNA damage and the percentage of lost and apoptotic cells. Results were considered significant with *p* ≤ 0.05.

## Results

### Selection of the treatment duration

**Fig. 1:**
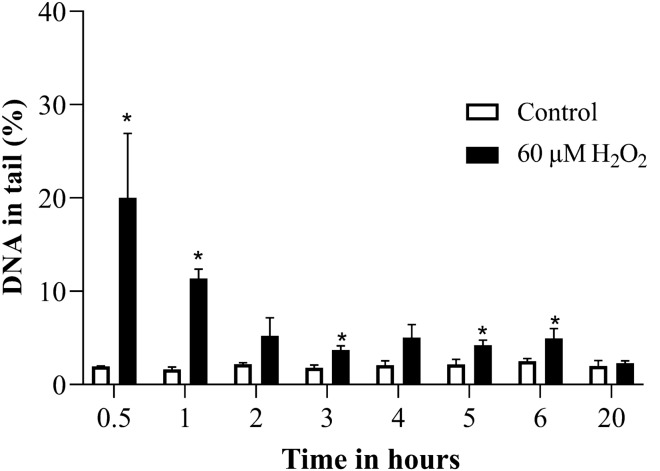
DNA strand breaks induced by 60 µM H_2_O_2_ over time in alkaline comet assay. *p ≤ 0.05 vs. Control at the same time point

TK6 cells were treated with one concentration of each substance (60 µM H_2_O_2_, 200 µM MMS and 1 µM etoposide), which was chosen according to our previous experience. The alkaline comet assay was performed with the treated cells and the solvent controls after 0.5, 1, 2, 3, 4, 5, 6, and 20 h. As can be seen in Fig. 1, H_2_O_2_ treatment yielded its maximum damage already after 0.5 h and the DNA strand breaks were reduced gradually over time after that, reaching control level after 20 h. Thus, 0.5 h was selected to test various concentrations of H_2_O_2_.

**Fig. 2:**
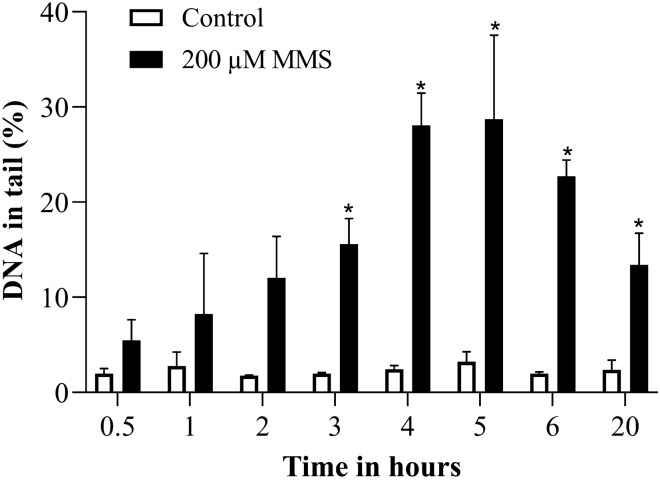
DNA strand breaks induced by 200 µM MMS over time in alkaline comet assay. *p ≤ 0.05 vs. Control at the same time point

MMS treatment induced a significant increase in DNA strand breaks from 3 h on. The maximally induced comet assay damage was reached at 4 to 5 h after the beginning of treatment, and then started to decline (Fig. 2). We selected the duration of 4 h for dose response experiments.

**Fig. 3:**
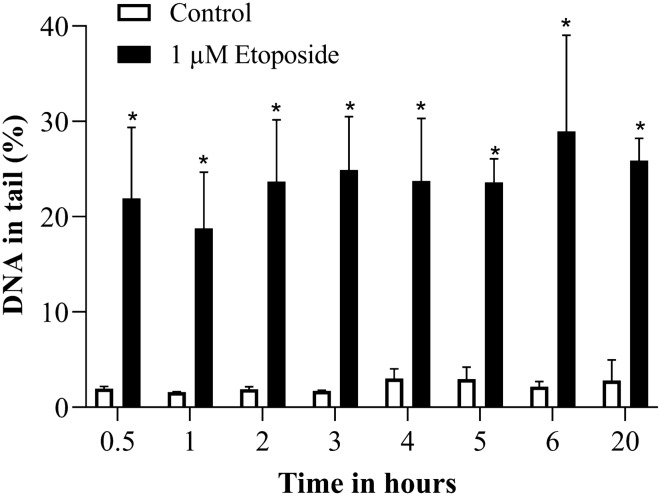
DNA strand breaks induced by 1 µM Etoposide over time in alkaline comet assay. *p ≤ 0.05 vs. Control at the same time point

Etoposide treatment induced a significant elevation already after 0.5 h treatment and the induced damage was more or less constant for the complete duration of observation (Fig. 3). From 5 h on, the number of ghost cells (in which DNA damage cannot be quantified) increased and scoring of the slides became difficult due to elevated background. Therefore, we selected the 4 h time point for further experiments.

### Concentration dependent increase in DNA damage

**Fig. 4:**
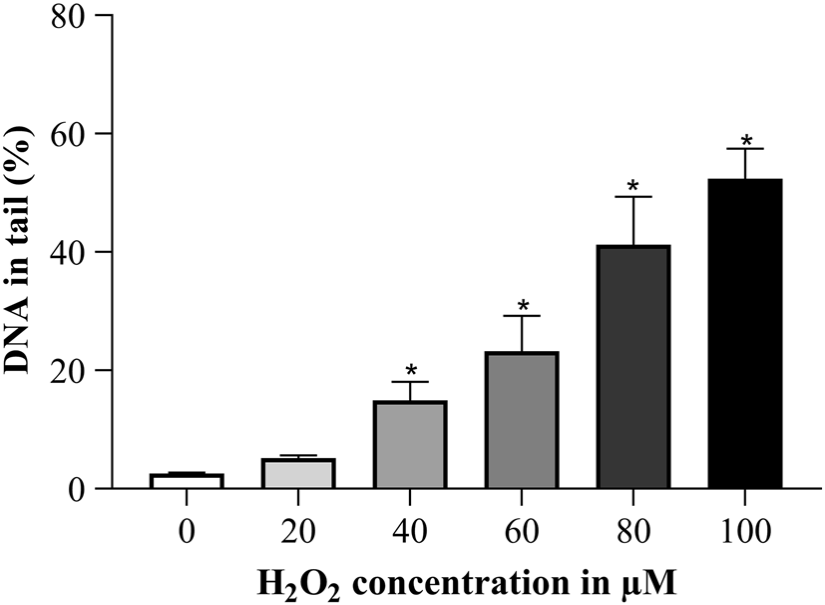
Concentration dependent increase in DNA damage after 0.5 h H_2_O_2_ treatment. *p ≤ 0.05 vs. 0 µM H_2_O_2_

H_2_O_2_ treatment was performed for 0.5 h with a concentration range from 20 to 100 µM. In Fig. 4, a concentration dependent increase in DNA damage can be seen and the elevation of DNA damage was significant with a concentration of 40 µM and more H_2_O_2_.

**Fig. 5:**
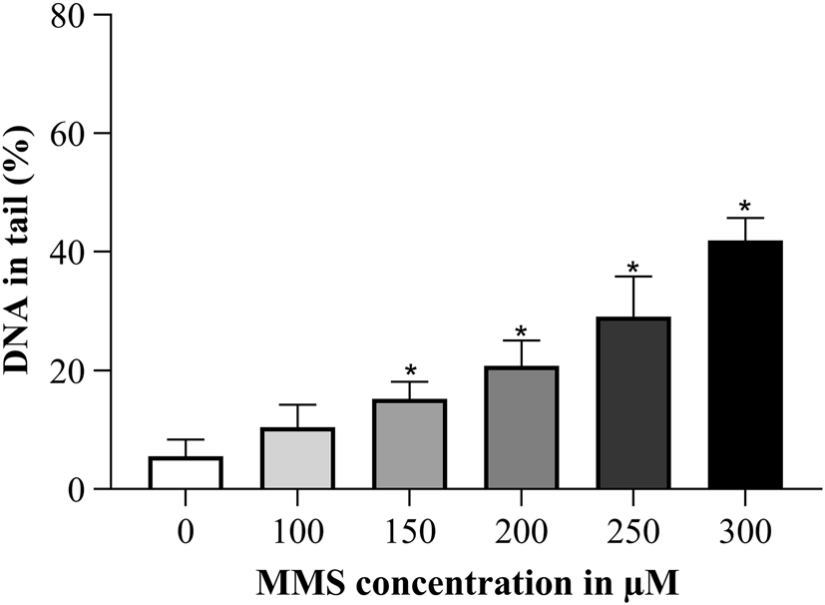
Concentration dependent increase in DNA damage after 4 h MMS treatment. *p ≤ 0.05 vs. 0 µM MMS

TK6 cells were treated with various MMS concentrations (0–300 µM) for 4 h and then the alkaline comet assay was performed. MMS treatment yielded a concentration dependent increase in DNA damage (Fig. 5) which was significantly higher than the control value at concentrations of 150 µM and more MMS.

**Fig. 6:**
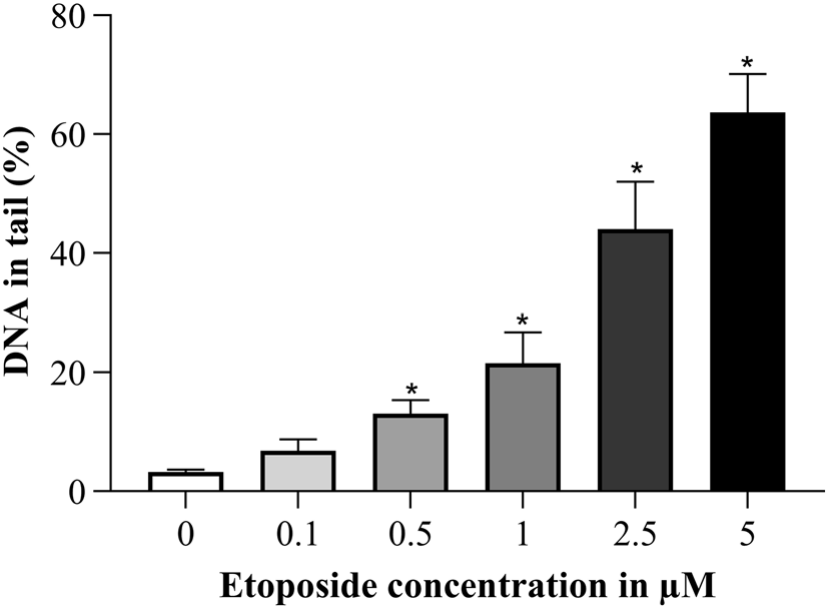
Concentration dependent increase in DNA damage after 4 h etoposide treatment. *p ≤ 0.05 vs. 0 µM etoposide

Etoposide treatment was performed for 4 h with a concentration range from 0.1 to 5 µM. The dose dependent increase was significantly elevated over control with a concentration of 0.5 µM and more etoposide (Fig. 6).

### Comparison of DNA damage levels and surviving cell number

In Table 1, the amount of DNA damage is shown together with the amount of disappeared, non-vital or apoptotic cells quantified on the next day. For assessment of cell number, to avoid a mixture of cell division and cell loss, the cytokinesis inhibitor cytochalasin B was added, preventing cell division after mitosis.

**Table 1:** DNA damage in relation to cell survival

Substance	Concentration (µM)	DNA in tail (%)	Non-vital cells (%) after 20 h (after subtraction of control)	Lost cells (%) after 20 h (after subtraction of control)	Apoptotic cells (%) after 20 h
H_2_O_2_	0	2.57 ± 0.21			0.95 ± 0.70
	20	5.20 ± 0.48	8.73 ± 3.10	6.82 ± 12.02	2.82 ± 2.30
	40	14.92 ± 0.48 (*)	15.90 ± 6.67 (*)	7.75 ± 22.93	27.00 ± 1.04 (*)
	60	23.23 ± 6.02 (*)	21.73 ± 2.78 (*)	14.39 ± 18.15	35.57 ± 2.74 (*)
	80	41.23 ± 8.14 (*)	25.40 ± 4.18 (*)	17.60 ± 17.62 (*)	43.50 ± 86.60 (*)
	100	52.41 ± 5.06 (*)	28.80 ± 0.66 (*)	21.04 ± 16.83 (*)	47.12 ± 13.74 (*)
MMS	0	5.57 ± 2.79			1.77 ± 0.80
	100	10.46 ± 3.84 (*)	3.57 ± 1.25 (*)	12.54 ± 0.47	3.93 ± 2.30
	150	15.22 ± 2.95 (*)	5.40 ± 2.35 (*)	13.49 ± 11.40	5.45 ± 2.80
	200	20.84 ± 4.30 (*)	8.67 ± 3.25 (*)	17.51 ± 13.71 (*)	10.80 ± 7.62
	250	29.15 ± 6.72 (*)	11.10 ± 3.14 (*)	26.25 ± 9.17 (*)	16.87 ± 10.52 (*)
	300	41.92 ± 3.84 (*)	14.27 ± 1 97 (*)	27.73 ± 17.75 (*)	26.53 ± 15.51 (*)
Etoposide	0	3.24 ± 0.41			1.50 ± 1.73
	0.1	6.83 ± 1.89	3.23 ± 0.93	25.13 ± 8.58 (*)	1.48 ± 0.76
	0.5	13.04 ± 2.30 (*)	12.67 ± 5.98 (*)	22.96 ± 2.47	14.37 ± 8.54 (*)
	1	21.52 ± 5.24 (*)	22.23 ± 2.37 (*)	28.81 ± 14.0 (*)	37.23 ± 12.20 (*)
	2.5	44.01 ± 8.02 (*)	19.17 ± 4.83 (*)	26.22 ± 3.09 (*)	61.12 ± 2.18 (*)
	5	63.64 ± 6.51 (*)	20.0 ± 5.50 (*)	14.78 ± 14.90	60.43 ± 3.47 (*)

Values for the percentage of dead cells and lost cells are shown after subtracting the control value

*p ≤ 0.05 vs. Control

The percentage of the lost cells after 20 h was evaluated according to following formula: $Lost cell \left(\%\right)after 20 h=\frac{[\left(\#Cell-0h*\%Vital cell-0h\right)-\left(\#Cell-20h*\%Vital cell-20h\right)]}{(\#Cell-0h*\%Vital cell-0h)}$

The data for the percentage of apoptotic cell were gained by the evaluation of slides prepared for the micronucleus test

For each substance, increasing concentrations led to an increase in dead cells (non-viable cells in the vitality test), lost cells (disappeared cell fraction from cell count) and apoptotic cells. To exclude the effect of proliferation, we used cytochalasin B, which still allows for nuclear division but prevents cell division. Therefore, cell number in this case reflects disappearance/loss of cells. Vitality was determined by assessment of cell membrane integrity and enzyme (esterase) activity. In principle cell membrane damage could be repairable, but that would be expected to occur shortly after substance exposure. If membrane leakage occurs one day later, it is highly likely a sign of cell death. This is then further supported by the absence of esterase activity. Apoptotic cells at the time of cell harvest, one day after exposure, were also determined. Substance induced apoptosis was at this time point probably mostly in a late apoptotic stage and such cells were most likely also detected in the viability assay as well because late stage apoptotic cells also have a compromised membrane integrity. However, the percentage of apoptotic cells was significantly higher than the percentage of dead cells with increasing H_2_O_2_ and etoposide concentrations. This difference between apoptotic and dead cells might be due to an increasing number of early stage apoptotic cells, which could be identified by their nuclear morphology but might have still had an intact cellular membrane with increasing H_2_O_2_ and etoposide concentrations.

### Correlation between DNA damage and cell survival

**Fig. 7:**
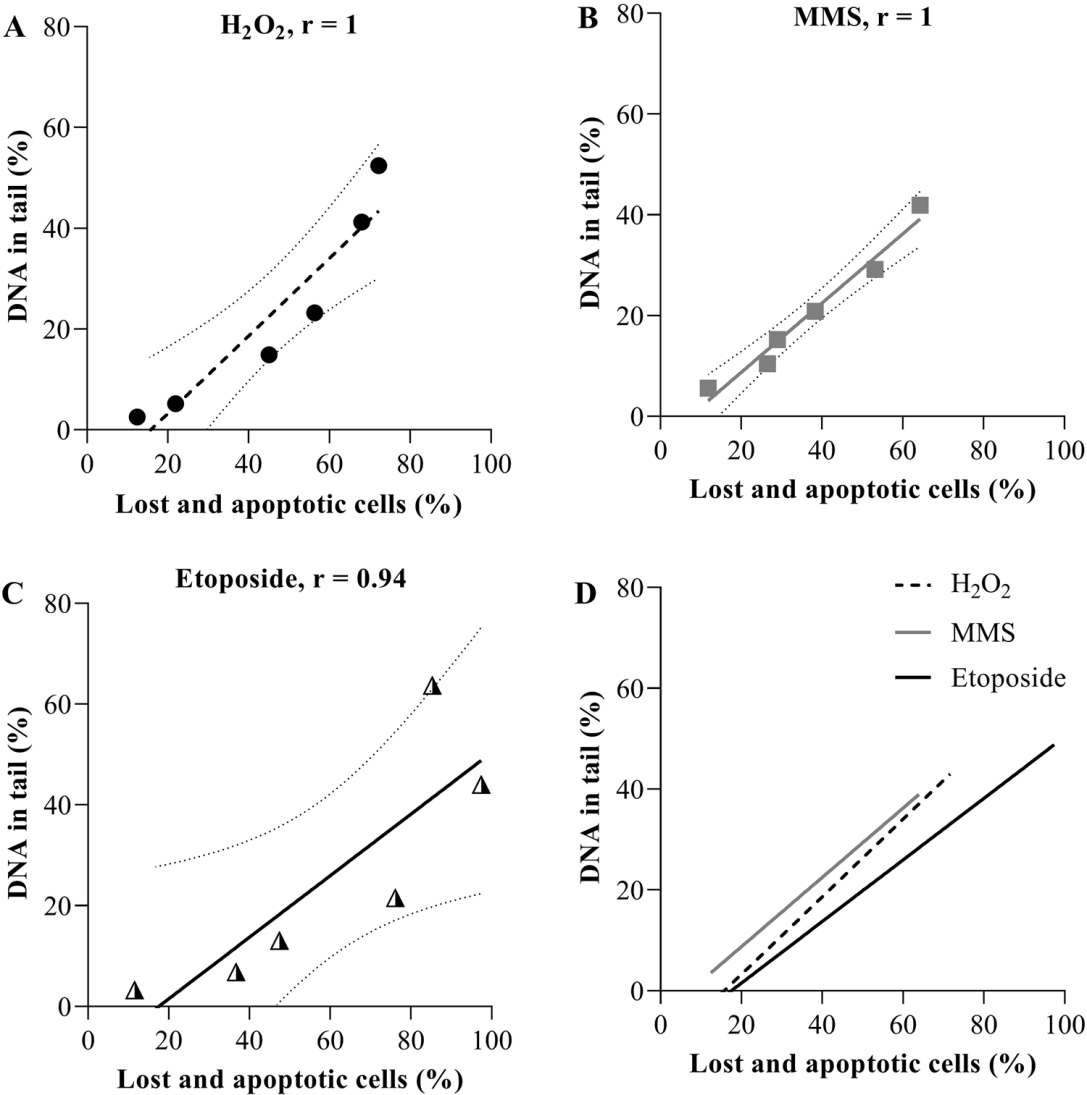
Correlation between the percentage of lost/apoptotic cells and the percentage of DNA in tail. (**A**) Correlation graph for H_2_O_2_ treatment. (**B**) Correlation graph for MMS treatment. (**C**) Correlation graph for etoposide treatment. (**D**) Correlation graphic for all three substances. Spearman correlation analysis was performed for each substance and the results showed a significant positive correlation between the percentage of lost/apoptotic cells and the percentage of DNA in tail

In Fig. 7, we added up the lost cells and the apoptotic cells to get an approximate idea regarding cell survival under these treatment conditions. Since we assume that the fraction of non-vital cells contained mostly late apoptotic cells, we did not add the non-vital cells separately. This number was correlated with the scored amount of DNA in tail for each of the three tested substances. The results from spearman correlation test indicate a strong positive correlation between the percentage of lost/apoptotic cells and increased DNA damage for all substances. A 50% reduced survival was reached with 56 µM H_2_O_2_, 241 µM MMS, and 0.8 µM etoposide (quantified using the % lost + apoptotic cells).

### DNA repair activity over time

**Fig. 8:**
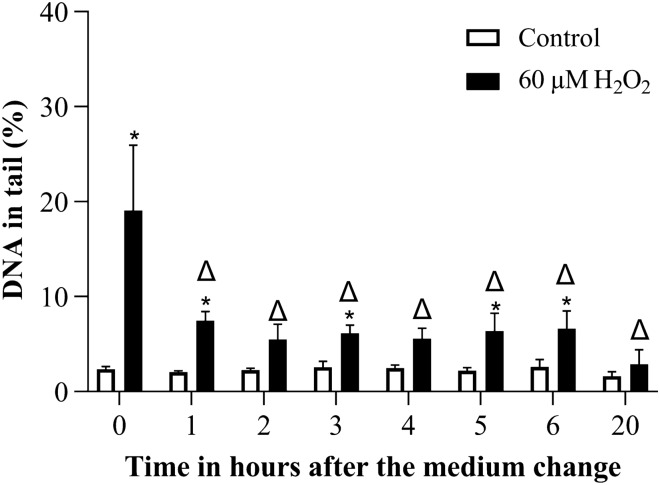
DNA damage reduction after H_2_O_2_ treatment over time in the comet assay. Treatment duration was 0.5 h and DNA damage was measured after medium change. *p ≤ 0.05 vs. Control at the same time point and ∆ ≤ 0.05 vs. 60 µM H_2_O_2_ at 0 h

Next, we intended to investigate whether cells are able to repair the DNA damage observed in the comet assay after removal of damaging agents. For this, we selected a concentration, which induced a clear DNA damage. In Fig. 8, DNA damage reduction following to 0.5 h H_2_O_2_ treatment is seen, which was significant within an hour after medium change and not different from the solvent control after 20 h.

**Fig. 9:**
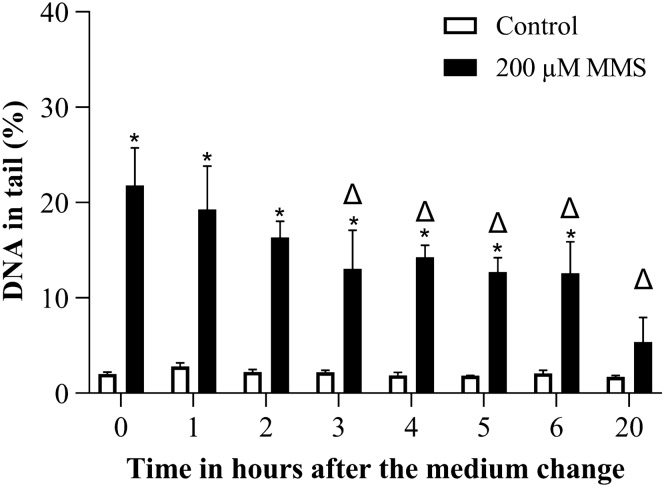
DNA damage reduction after MMS treatment over time in the comet assay. Treatment duration was 4 h and DNA damage was measured after the medium change. *p ≤ 0.05 vs. Control at the same time point and ∆ ≤ 0.05 vs. 200 µM MMS at 0 h

The DNA damage induced by MMS treatment was significantly reduced 3 h after the medium change and was not significantly higher than the control after 20 h (Fig. 9).

**Fig. 10:**
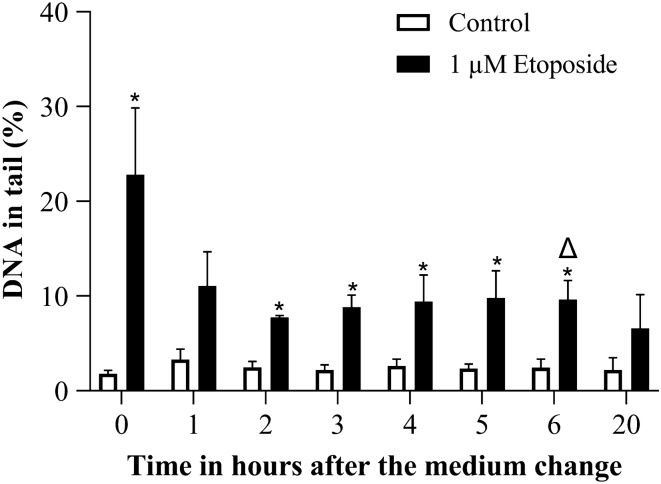
DNA damage reduction after etoposide treatment over time in the comet assay. Treatment duration was 4 h and DNA damage was measured after the medium change. *p ≤ 0.05 vs. Control at the same time point and ∆ ≤ 0.05 vs. 1 µM etoposide at 0 h

We observed a clear reduction in DNA damage induced by etoposide treatment 1 h after the medium change. This reduction remained almost the same over time with small fluctuations. The reduction in etoposide induced DNA damage was significant compared to directly after treatment after 6 h, and the remaining DNA damage was not significantly different from the control at 20 h (Fig. 10).

If DNA repair is not successful, gene or chromosomal mutations may result. Chromosomal mutations can be detected as micronuclei. Therefore, we analyzed the frequency of micronuclei at the time of harvest (Table 2).

**Table 2:** Micronucleus (MN) frequency, proliferation index (CBPI), and the percentage of healthy proliferating cells

Substance	Concentration (µM)	# MN cells (%)	CBPI	Proliferating cell (BN + MuN) (%)
H_2_O_2_	0	1.37 ± 0.48	2.04 ± 0.10	74.87 ± 13.60
	20	9.35 ± 2.68 (*)	1.79 ± 0.06 (*)	59.73 ± 4.61 (*)
	40	9.98 ± 1.01 (*)	1.64 ± 0.11 (*)	35.42 ± 2.20 (*)
	60	8.73 ± 1.73 (*)	1.63 ± 0.04 (*)	31.15 ± 2.20 (*)
	80	8.70 ± 1.78 (*)	1.51 ± 0.19 (*)	24.73 ± 7.16 (*)
	100	8.97 ± 2.66 (*)	1.45 ± 0.20 (*)	22.70 ± 9.07 (*)
MMS	0	1.45 ± 0.6	2.00 ± 0.03	81.42 ± 3.67
	100	3.12 ± 0.45 (*)	1.83 ± 0.05 (*)	71.28 ± 5.97 (*)
	150	4 48 ± 0.51 (*)	1.78 ± 0.06 (*)	64.60 ± 4.08 (*)
	200	6.37 ± 1.05 (*)	1.63 ± 0.04 (*)	49.98 ± 6.49 (*)
	250	7.97 ± 1.00 (*)	1.61 ± 0.05 (*)	42.12 ± 6.82 (*)
	300	8.42 ± 1.27 (*)	1.60 ± 0.1 (*)	38.02 ± 3.02 (*)
Etoposide	0	1.57 ± 0.65	1.96 ± 0.05	77.92 ± 5.47
	0.1	10.15 ± 1.10 (*)	1.83 ± 0.11 (*)	62.15 ± 7.40 (*)
	0.5	6.45 ± 3.51 (*)	1.38 ± 0.07 (*)	23.00 ± 4.07 (*)
	1	n.d	1.23 ± 0.03 (*)	10.48 ± 2.69 (*)
	2.5	n.d	1.08 ± 0.02 (*)	2.95 ± 0.78 (*)
	5	n.d	1.07 ± 0.01 (*)	2.53 ± 0.56 (*)

Healthy proliferating cells are percent binucleated (BN) plus multinucleated (MuN) cells. n.d. (not determined) number of binucleated cells was not sufficient for MN-evaluation

* *p* ≤ 0.05 vs. Control (0 µM)

H_2_O_2_ induced a significant induction in micronucleus frequency with all applied concentrations, which reached a plateau at concentrations of 20 µM and higher. MMS led to a concentration dependent significant increase in micronucleus formation. Since etoposide treatment reduced the cytokinesis block proliferation index to a large degree from 1 µM on, micronucleus frequency could only be quantified for the lower concentrations, and was significantly different compared to control cells. For all substances, we observed a concentration dependent decrease in the cytokinesis block proliferation index and the percentage of proliferating cells. Since micronuclei are a subtype of chromosomal aberrations and are considered as an end-point for chromosomal mutation, our results showed that DNA damage determined by comet assay was associated with a mutagenic damage in the same cell culture.

## Discussion

Our motivation for this study was to develop a better understanding for the meaning of DNA damage as detected in the comet assay. DNA damage in the comet assay in in vitro experiments can range up to 30 or even 50% of DNA in tail. With that much cellular DNA affected, the question is whether cells harboring comet assay damage are death-prone, whether the damage can be repaired, or whether both is possible, depending on the amount of DNA damage.

For this aim, we used human lymphoblastoid TK6 cells and treated them with three substances inducing DNA damage with different mechanisms. H_2_O_2_ was selected as a reactive oxygen species causing DNA base oxidation and strand breaks (Halliwell et al. 2000), MMS as a DNA alkylating agent (Beranek 1990) and etoposide as a topoisomerase II inhibitor (Baldwin and Osheroff 2005; Montecucco and Biamonti 2007). The maximally induced DNA damage was chosen as suitable end-point for quantification. To determine the required treatment duration for maximal effect, we first analyzed a time course of damage induction in the comet assay. H_2_O_2_ as highly reactive substance induced maximal damage within 0.5 h, while MMS-induced DNA damage reached its maximum at 4 h and etoposide induced DNA damage quickly reached a plateau with some fluctuation. However, the presence of many ghost cells at later time points suggested also 4 h as optimal time point for etoposide.

The dose response relationships then showed a dose dependent increase of DNA damage for each substance as expected. When we correlated the DNA damage with cell survival, we found that with higher DNA damage less viable and non-apoptotic cells were still present on the next day. The substance concentrations for reduction to 50% survival were orders of magnitude different with etoposide < H_2_O_2_ < MMS. This is due to their mechanism of action, but also to their stability in medium and their ability to reach their target despite the presence of FCS in the medium and many molecules to react with inside the cells (Faheina-Martins et al. 2011). However, the focus here was the relationship between amount of DNA in tail and loss of cells. If the number of DNA breaks and other lesions that are detectable in the comet assay would be the only determinant of cell survival, one would expect the same relationship between amount of DNA in tail and loss of viable cells for all substances. However, while 25–30% of DNA in tail as maximally induced damage was needed to cause the loss or death of half of the cells for H_2_O_2_ and MMS, only 20% of DNA damage was sufficient for the same amount of cell loss and death after etoposide treatment. Clearly, not only the induced DNA damage can lead to cell death, but other cellular targets for toxicity also contribute. For example, the highly reactive H_2_O_2_ is known to oxidize proteins and lipids (Halliwell et al. 2000). Similarly, MMS can alkylate cellular macromolecules besides DNA (Yang and Bartlett 2016; Zhang et al. 2005). Etoposide is thought to act specifically on topoisomerase II, which not only leads to strand break formation, but to cell cycle arrest resulting in cell death if the arrest cannot be overcome (Clifford et al. 2003; Schonn et al. 2010). Nevertheless, and across all three tested substances, it can be stated that even percentages of more than 10% DNA in tail do result in considerable cell death (20% or more) later on.

Next, we investigated whether the DNA damage detected in the comet assay can be repaired. For this, we exchanged the medium after treatment and followed the cells over time. H_2_O_2_ induced damage and etoposide induced damage were reduced to about half within an hour and then reduced to non-significant difference from control until the next day. MMS-induced damage was only gradually reduced, reaching about half of the initial values at 3 h, and was not significantly different from control any more on the next day. The results for H_2_O_2_ and etoposide are well in line with published literature, while more variation is observed for MMS. Duthie and Collins, (Duthie and Collins 1997), measured the DNA repair activity up to an hour in HeLa cells following to 30 min H_2_O_2_ treatment on ice. Their findings indicated a quick repair of H_2_O_2_ induced damage that was almost completely reduced within an hour after the treatment. Benhusein et al. (2010), treated HepG2 cells with H_2_O_2_ for 5 min, 30 min, 40 min, 1 h and 24 h. Their findings showed a significant increase in DNA damage in the comet assay after 5 min, which reached its maximum at 1 h. However, the DNA damage after 24 h was not different from the control. Ngo et al. (2021), found a halftime for repair of H_2_O_2_ induced DNA damage in the comet assay of 24 min for TK6 cells and 39 min for a human lymphocyte sample. Regarding etoposide, Schonn et al. (2010), found in human colon cancer a significant repair of etoposide induced DNA damage within 1.5 h in the comet assay. With MMS, Valdiglesias et al. (2020), found that already within one hour, half of MMS-induced % tail in DNA war repaired in fresh human blood samples. In V79 cells, reduction of the damage to half was reached between 2 and 4 h (Viau et al. 2009). Possibly, the cellular response to MMS depends more on the cell line and the applied concentration than for the other two compounds.

In principle, DNA damage reduction over time may be due to DNA repair or to the loss of heavily damage cells by cell death. The first few hours of damage reduction are most likely due to DNA repair because loss of cells through apoptosis or other forms of cell death requires at least several hours. In the case of longer treatment, durations of 4 h (MMS and etoposide) cell death initiated at the beginning of treatment may already contribute to damage reduction within the observation time on the same day. Reduction of DNA damage on the next day might have been due to lost cells in addition to DNA repair. Overall, the percentage of reduction in DNA tail over time was most likely partly due to repair and partly due to cell death. This is an important aspect to consider during the interpretation of variants of the comet assay designed to measure DNA repair. There are various modifications of the comet assay, which can be utilized as DNA repair end-point. The most popular ones are to follow the reduction of DNA damage over time (e.g. Cebulska-Wasilewska 2003; Lorenzo et al. 2008)) or the aphidicolin block repair assay (e.g. Azqueta et al. 2014; Speit et al. 2013; Vande Loock et al. 2010)). In this latter variant, blocking repair polymerase alpha leads to accumulation of DNA breaks after the incision activity (Collins 2004). As long as both methods analyze a short time frame of a few hours after the induction of lesions they most likely measure DNA repair, but longer treatment or observation periods might yield less clear results. A special situation is the measurement of freshly thawed lymphocytes, in which cell death and concurrent elevation and reduction of DNA damage may occur within hours (Bankoglu et al. 2021) and require special concern if DNA repair is to be analyzed. The third popular version of measuring DNA repair activity using comet assay is to use a cellular extract containing repair enzymes. The extract is then incubated with substrate cells harboring specifically induced lesions (Vodenkova et al. 2020). In this variation, any excision of lesions must be due to DNA repair. However, this variation requires a high number of cells for extract preparation (Collins and Azqueta 2012).

MMS-induced lesions were repaired more slowly than H_2_O_2_ and etoposide induced lesions. H_2_O_2_ treatment can directly induce DNA strand breaks as well as formation of oxidized purines (Poetsch 2020). Oxidized purines such as 8-oxodG can be converted to apurinic (AP) sites, which are prone to single strand break formation, which is also further enabled during the comet assay procedure (Cappelli et al. 2000; Gorini et al. 2021). Thus, H_2_O_2_ induced lesions are subject to base excision and partly to nucleotide excision (Dizdaroglu et al. 2017). MMS-induced alkylation of DNA bases can cause apurinic sites, strand breaks (due to closely located excision repair sites), cell cycle arrest and cell death (Fu et al. 2012). They can also block replication fork elongation, causing formation of replication-associated DNA lesions, likely double-strand breaks (Groth et al. 2010). Thus, MMS-induced lesions are in principle subject to the same repair mechanisms as H_2_O_2_ induced lesions (Fu et al. 2012), but the MMS-induced burden on the cells is more persistent due to a delayed additional induction of highly toxic DSB (Ensminger et al. 2014), which explains the slower repair kinetics. Etoposide inhibits topoisomerase II by stabilizing the cleavable complex formed between the enzyme and the DNA strands that is cuts and reseals (Sun et al. 2020). It interacts at the enzyme–DNA interface in a noncovalent manner (Smith et al. 2014). During the comet assay procedure, the enzyme is detached from the DNA and strand breaks remain. If etoposide is washed off by medium exchange, topoisomerase II can continue its task and reseal the breaks within the cleavable complex without the need for additional DNA repair system. Oxidative etoposide metabolites such as etoposide quinone may bind covalently to the cleavable complex and remain in the cell to cause further damage. However, TK6 cells exhibit negligible amounts of cytochrome P450 enzymes, which oxidize etoposide to its quinone (Li et al. 2020; Shah et al. 2016). If the cleavable complex persists (in case etoposide is not washed out or is not completely removed), it interferes with cellular processes such as replication and transcription and the cell attempts to remove topoisomerase II and to repair the remaining DNA double-strand breaks predominantly by NHEJ (Montecucco et al. 2015).

Another question was whether the treatment inducing comet formation also induces heritable mutations. Micronucleus formation is a well-accepted end-point for genomic instability and chromosomal mutagenesis. Micronuclei can form from lagging whole chromosome or acentric chromosome fragment, which do not incorporate to one of the daughter nuclei during cell division (Fenech 2007). After H_2_O_2_ treatment, we observed a significant increase in micronucleus formation already with the lowest selected concentration. The micronucleus frequency after MMS treatment was concentration dependent. Micronucleus formation after etoposide treatment could only be scored with the lowest applied concentrations due to the proliferation inhibiting effect of higher concentrations. Thus, even the treatments causing less than 15% DNA in tail did induce chromosomal mutations.

A limitation of the present study is that it only investigates one cell line. It is likely that the consequences of DNA damage for the future fate of a cell depends on its genomic composition, i.e. mutations in DNA-damage-repair related genes, which might be harbored by permanent cultured cell lines. TK6 cells were chosen because they do have a wild type p53 gene, and are very frequently used in mutagenicity testing. However, we expect that other cell types might react differently. While some tumor cell lines overexpress certain DNA repair genes (Erasimus et al. 2016; Zhang et al. 2021), stem cells are thought to react sensitively with cell death to DNA damage (Weeden and Asselin-Labat 2018). Whereas cell lines or primary lymphocytes are used for routine mutagenicity testing, tumors arise from mutations in stem cells and the tumor cells are relevant for identification of sensitivity towards chemotherapeutic DNA-damaging agents. Considering all of these aspects, it is important to follow-up both in further research.

Although the amount of cell death relative to DNA damage depended on the test substance, for all three tested substances DNA damage levels greater than about 20–30% lead to death of more than half of the treated cells. At DNA damage levels lower than 15% in the comet assay, these tested agents still induced a significant increase in micronucleus formation. Therefore, we consider this DNA damage range of up to 15% DNA in tail as more reliable regarding interpretation of achieved results than DNA tail values in the higher range. This range of less than 15% DNA in tail is mostly measured in human biomonitoring studies, which supports the validity of results obtained with the comet assay in this application further. For in vitro experiments including higher than 20% DNA tail values it is recommended to present cell survival data in parallel.

## Data Availability

Available upon request.
